# Glycosylation of the retina

**DOI:** 10.1042/BST20260004

**Published:** 2026-09-02

**Authors:** Jaclyn Swan, Pascal Gagneux

**Affiliations:** 1 Department of Ecological, Plant and Animal Science, School of Agriculture, Biomedicine and Environment, La Trobe University, Bundoora, Australia; 2 Department of Pathology, University of California San Diego, La Jolla, California, U.S.A.; 3 Department of Anthropology, University of California San Diego, La Jolla, California, U.S.A.; 4 Center for Academic Research and Training in Anthropogeny (CARTA), La Jolla, California, U.S.A.

**Keywords:** glycan, glycobiology, lectin, post translational modification

## Abstract

Unlike the cornea and tear fluid, glycosylation within the retina remains remarkably underexplored. This review highlights the diverse roles of glycans in retinal development and vision, with a focus on major glycan classes, including glycosphingolipids (GSLs; glycans attached to lipids), N- and O-linked glycans on proteins, glycosaminoglycans (GAGs) on proteoglycans, O-mannosylated proteoglycans, and intracellular O-GlcNAcylation. Together, these components provide a comprehensive framework for understanding glycan-mediated processes in retinal biology. Current knowledge of the retinal glycosylation nanolandscape, primarily derived from lectin binding and anti-glycan antibody studies, is summarised. The review also examines the roles of altered glycosylation in immune regulation and retinal diseases such as retinitis pigmentosa, age-related macular degeneration and diabetic retinopathy. Finally, key gaps in knowledge and priority areas for future research are discussed. The retina remains understudied with regard to the complex patterns of glycosylation. Alterations of these patterns could represent consequences or be driving pathology, given the well-established roles of glycan patterns in development and homeostasis in other tissues.

## Introduction

Vision impairment is a frequent consequence in congenital disorders of glycosylation and highlights the essential role of glycan-dependent processes in eye development and the maintenance of normal retinal function [1]. Glycans are complex, often heteropolymeric carbohydrates attached to proteins and lipids that regulate protein folding, stability, trafficking, extracellular matrix organisation, and cell–cell communication through interactions with glycan-binding proteins such as lectins [2]. As a major form of post-translational modification, glycosylation is therefore critical within the highly specialised retinal environment. While glycan structure and function have been relatively well characterised in tear fluid and the cornea, where glycans provide lubrication and barrier protection, glycosylation within the retina remains comparatively underexplored [3–7]. One plant lectin, peanut agglutin (PNA), which specifically binds to the disaccharide sequence Galβ1–3GalNAc (galactose-beta1,3-N-acetylgalactosamine) on glycoconjugates, is widely used as species-universal marker of specific retinal cell populations [8], highlighting the conserved nature of glycan motifs across vertebrate retinas. Despite their utility in cell identification, many retinal glycans remain functionally uncharacterised, representing a key gap in understanding glycosylation in retinal biology and disease.

This gap is critical given the retina’s limited regenerative capacity, as most neurons fail to recover after injury or degeneration [9]. Nevertheless, retinal cells retain the ability to dynamically remodel their glycosylation profiles in response to developmental cues, environmental changes, and pathological stress. Glycan expression is tightly regulated during retinal development, contributing to cell fate specification, synaptic refinement, and circuit formation [10]. In the mature retina, such dynamic glycosylation changes may contribute to mechanisms of neural plasticity, enabling cells to modulate interactions without requiring proliferation or replacement. Glycosylation also plays a central role in maintaining retinal immune homeostasis by regulating complement activation, microglial recognition, and the balance between debris clearance and inflammatory responses [11–15]. Beyond congenital disorders of glycosylation that directly impair vision [1], there is currently limited evidence that altered glycosylation is a primary driver of retinal disease; however, numerous glycosylation changes have been associated with retinal disorders, including retinitis pigmentosa (RP), age-related macular degeneration (AMD), and diabetic retinopathy (DR). Given that vision depends on a finely tuned immune balance, understanding how glycosylation governs immune signalling and intercellular communication in the retina is essential.

## Retina structure

**Figure 1:**
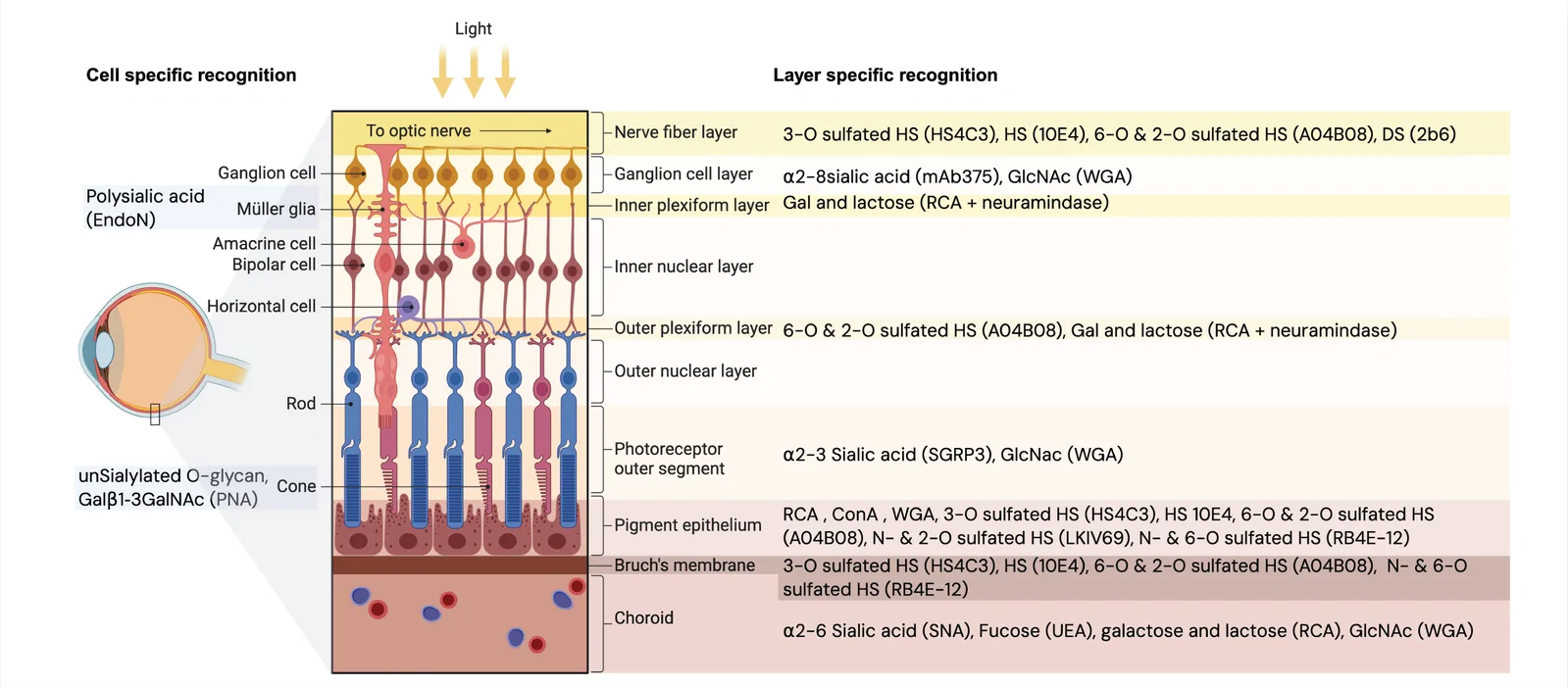
Spatial distribution of glycan motifs across human retinal cell layers Schematic of the human eye showing a cross-section of the retina and the cells that make up each layer. The glycan ligand and (lectin/antibody/phage) that binds it, collated from a number of previous studies, are indicated for each layer or cell type [19–24]. Created in BioRender. Swan, J. (2026) https://BioRender.com/936nrza

The retina is a highly specialised and exquisitely organised tissue composed of distinct cell types arranged into well-defined layers (Figure 1). Together, these cells detect incoming photons and convert them into electrical signals that are transmitted to and interpreted by the brain [16]. The innermost retinal layer contains retinal ganglion cells, whose axons converge to form the optic nerve. Adjacent is the inner plexiform layer, where ganglion cells form synapses with interneurons, including amacrine, bipolar, and horizontal cells [16]. The nuclei of these interneurons constitute the inner nuclear layer, while their synaptic connections with photoreceptors occur within the outer plexiform layer [17]. Photoreceptors, both rods and cones, are located in the outer retina, with their nuclei forming the outer nuclear layer and their light-sensitive outer segments comprising the photoreceptor layer. Supporting the photoreceptors is the retinal pigment epithelium (RPE), a monolayer of specialised and pigmented epithelial cells essential for photoreceptor survival, outer segment renewal, and metabolic support. Because photoreceptors lack direct access to the retinal vasculature, they rely on the RPE and the underlying choroidal circulation for nutrient and oxygen supply [16]. Similar to the central nervous system, the retina is an immune-privileged tissue [18]. The blood–retina barrier is maintained by tight junctions between RPE cells, in conjunction with Bruch's membrane and the choroidal vasculature, thereby tightly regulating molecular and immune cell access to the neural retina.

## Glycans in vison

### N- and O-glycans of the retina

N- and O-glycans are carbohydrate modifications on glycoproteins. N-glycans share a conserved core, are transferred en bloc onto nascent polypeptides in the endoplasmic reticulum (ER) [2], then remodelled in the ER and Golgi by glycosidases and glycosyltransferases to generate high-mannose, hybrid, or complex structures. In contrast, O-glycans lack a conserved core and are initiated in the Golgi by stepwise addition of N-acetylgalactosamine (GalNAc) to serine or threonine residues, followed by elongation, to produce diverse structures [2].

Opsins and rhodopsin are integral membrane proteins that define the outer segments of cone and rod photoreceptors, respectively. Cone opsins mediate high-acuity, colour, and central vision, whereas rhodopsin in rod photoreceptors underpins scotopic, low-light, and peripheral vision [25]. All known variants of these photopigments are glycosylated, carrying N-linked, O-linked glycans, or a combination thereof [26–28]. While N-glycosylation of opsins and rhodopsin does not appear to be strictly required for targeting or localisation to the photoreceptor outer segment, it plays a critical role in regulating protein turnover and maintaining photoreceptor homeostasis. Disruption of glycosylation can lead to abnormal protein processing and increased degradation. Mutations that abolish N-glycosylation N12 site in human rhodopsin expressed in transgenic *Xenopus laevis* cause light-sensitive retinal degeneration, underscoring the essential role of glycan modifications in preserving photoreceptor survival and visual function [29,30].

Glycosylation is also critical for other outer segment proteins. The essential transmembrane protein retinal degeneration slow (RDS; also known as peripherin-2) is expressed in the outer segments of both rod and cone photoreceptors, and supports disc morphogenesis and structural stability [31]. Mutations in the *RDS* gene are associated with a range of inherited retinal degenerations. Notably, RDS contains a conserved N-glycosylation site that is essential for heterodimer oligomerisation with its homolog ROM-1, demonstrated in a mouse knock-in model [31]. The glycan ROM-1 interaction stabilises RDS complexes and is required for maintaining photoreceptor structure and function [31], underscoring a context-dependent role for glycosylation in regulating protein–protein interactions within the outer segment.

### Proteoglycans/glycosaminoglycans

Glycosaminoglycans (GAGs) are linear polysaccharides composed of repeating disaccharide units with variable sulfation, generating substantial structural and functional diversity. GAGs are generally attached to core proteins, forming proteoglycans that are major components of the extracellular matrix. GAGs are classified into several major families, including heparan sulphate (HS), chondroitin sulphate (CS), dermatan sulphate (DS), keratan sulphate (KS), and hyaluronan (HA). HS is characterised by highly variable sulfation and plays key roles in growth factor signalling and gradient formation, is a major component of the Bruch’s membrane [32,33]. CS and DS differ in uronic acid composition and epimerisation, contributing to distinct roles in structural support and cell–matrix interactions. KS lacks uronic acid and is associated with corneal transparency and neural tissues [34,35]. Unlike other GAGs, HA is a non-sulfated, high-molecular-weight polydisaccharide synthesised at the cell membrane that is not covalently linked to a core protein via a tetrasaccharide linker but instead organises extracellular matrix architecture through cross-linking by hyaluronan-binding proteins such as CD44 and HABP1/2 [36,37]. Topically hyaluronan was first identified in the vitreous [38].

Within the retina, proteoglycans are critical components of the interphotoreceptor matrix (IPM), the specialised extracellular matrix surrounding photoreceptors. IPM proteoglycans, including IMPG1 and IMPG2, are major constituents of this niche and contain N-, O-, and GAG glycosylation [39]. Mouse knockout models have demonstrated that N-terminal N-glycosylation of these proteoglycans is essential for maintaining photoreceptor function, highlighting the importance of glycosylation in preserving retinal integrity [39]. More broadly, proteoglycans and their associated GAG chains play fundamental roles in vision by regulating cell adhesion, signal transduction, and extracellular matrix support, as extensively reviewed by Melrose [40].

### Glycosphingolipids

Among glycolipids, glycosphingolipids (GSLs) represent the predominant class in vertebrates and are key components of cellular membranes. GSLs consist of glycan structures assembled stepwise onto a ceramide backbone. GSLs preferentially localise to membrane microdomains, where they contribute to the formation of lipid rafts that organise signalling complexes and regulate membrane dynamics [41]. A major subclass of GSLs, gangliosides, are defined by the presence of terminal sialic acid residues and are particularly abundant in neural tissues, where they play important roles in neuronal development, synaptic function, and neurodegenerative processes [41,42]. GT3, a trisialylated ganglioside, is currently used as telltale marker for neuronal progenitor cells and is recognised by the monoclonal antibody A2B5 [43], and the neuronal marker NCAM is visualised by a monoclonal antibody mAB735 that recognises polysialic acid [44].

Within the retina, emerging evidence indicates that GSLs contribute to both structural integrity and cellular communication. GSLs such as GD3 are thought to support adhesion between the neural retina and the RPE, an interaction essential for photoreceptor survival [45,46]. Several ganglioside species have been identified in the human retina, and functional studies in knockout mouse models have demonstrated that specific gangliosides, such as GD3, are required for maintaining retinal structure and visual function [45]. The sialic acid residues of gangliosides have been implicated in mediating neural retinal adhesion in an *in vitro* cell assay using embryonic chick neural retinal cells, further highlighting the importance of GSL-mediated interactions in maintaining retinal architecture and function [47].

### O-Mannose

Like O-glycans, O-mannose glycans are attached to the serine or threonine via an O-linkage; however, the initial monosaccharide is a mannose residue instead of GalNAc. O-mannosylation is essential for development and growth and represents the glycan class most strongly associated with congenital disorders of glycosylation. This modification is initiated in the ER by the transfer of mannose to serine/threonine residues by protein O-mannosyltransferases (POMGNT1/2), followed by subsequent glycan elongation. In the retina, POMGNT1 is expressed in photoreceptors, and its dysfunction disrupts the assembly and organisation of the inner limiting membrane within the neural retina [48]. Interactions between HS proteoglycans and O-mannosylated dystroglycan are critical for phototransduction and synaptic transmission, facilitating signal transfer between photoreceptors and bipolar cells [49].

### O-GlcNAc

O-GlcNAcylation is a post-translational modification involving the addition of a single GlcNAc moiety to hydroxyl of serine or threonine residues of intracellular proteins [50]. Unlike classical O-glycosylation, which is initiated by any of a large number of GalNAc transferases and is often extended into more complex glycan structures, O-GlcNAcylation consists of a single sugar modification and catalysed by a single enzyme. Functionally, O-GlcNAc is analogous to phosphorylation in that it is highly dynamic [51]; this and its intracellular location make O-GlcNAcylation unique compared with the glycans described above. O-GlcNAc has been identified on over 1000 proteins and is involved in a wide range of cellular processes [50]. The addition of O-GlcNAc is catalysed by O-GlcNAc transferase (OGT), while its removal is mediated by O-GlcNAcase (OGA), enabling rapid and reversible regulation known as O-GlcNAc cycling [2].

In the retina, >60 proteins known to be O-GlcNAcylated in other tissues are also expressed [50], and a subset has been directly implicated in retinal disease [52–54]. Dysregulation of O-GlcNAcylation has been linked to DR (see section: Diabetic Retinopathy). More broadly, these findings highlight the importance of tightly controlled O-GlcNAc cycling for maintaining retinal homeostasis. Excess O-GlcNAcylation has been associated with vascular dysfunction [52], and increased O-GlcNAc levels have also been observed in the ganglion cell layer and inner nuclear layer, where they promote nuclear translocation of the RelA subunit of the nuclear factor-kappa B (NF-κB) complex and enhance NF-κB transcriptional activity, a pathway that may contribute to retinal ganglion cell apoptosis [55]. In addition, pharmacological inhibition of OGA using Thiamet-G, which prevents O-GlcNAc from being removed from protein, results in increased mitochondrial activity in mouse models [53], and shown to increase Cd40 mRNA expression in Müller glial cells thought to increase retinal inflammation [54]. Together, these observations reinforce the role of O-GlcNAcylation as a key regulator of retinal physiology and pathology, particularly in the context of metabolic stress.

## Glycan landscape of the human retina: lectin and antibody analysis

Building on the structural and functional roles of major glycan classes, lectin- and antibody-based approaches have provided key insight into the spatial organisation of glycans within the human retina. Commercially available and laboratory-developed lectins have been widely used to characterise glycosylation patterns, revealing both conserved and highly specialised glycan distributions across retinal layers. While some motifs, such as oligomannose [23] and the conserved chitobiose core (GlcNAcβ1–4GlcNAc) [23,56,57], are widely detected but most abundant in the photoreceptor layer, other lectins identify strong cell type–specific localisation, reflecting tightly regulated glycan expression within the neural retina (Figure 1).

The photoreceptor layer displays a distinct glycosylation profile enriched in α2–3-linked sialic acid [22] and galactose residues. PNA binds Galβ1–3GalNAc O-glycans and is a well-established marker of cone photoreceptors across species [8,57], while its binding to rods is only observed following sialidase treatment, indicating masking by terminal sialic acids in healthy tissue [23]. Intravitreal PNA labelling does not impair visual function [58], supporting that photoreceptor O-glycans are specialised, non-sialylated in cones and predominantly α2–3 sialylated in rods, although their functional roles remain unclear.

Distinct glycosylation signatures are also observed within the retinal vasculature, where endothelial cells exhibit glycan profiles that differ markedly from the surrounding neural tissue. These vascular-associated glycans, characterised by terminal fucosylation and α2-6-linked sialic acid [19,22], form discrete punctate structures throughout the inner retinal layers, reflecting the molecular specialisation of vascular compartments within the retina.

More recently, modified endoneuraminidase (EndoN)-based probes have been suggested as markers of Müller glial cells [24], while the monoclonal antibody mAb735 has demonstrated polysialic acid binding within the ganglion cell layer and an irregular distribution in the choroid [22]. Notably, binding to polysialic acid is abolished following treatment with active EndoN, confirming specificity. These observations highlight the emerging utility of targeted glycan detection approaches for resolving cell type–specific glycosylation patterns within the retina.

Certain glycan features are tightly restricted, exemplified by the absence of N-glycolylneuraminic acid (Neu5Gc) from human neural tissues, including the retina. Consistently, Neu5Gc is undetectable in human retinal tissue by DMB-HPLC and shows no antibody recognition outside the vasculature [22]. Preliminary evidence suggests this exclusion may extend beyond humans, indicating a potentially evolutionarily conserved constraint within neural tissues [59].

Notably, many lectin-based studies rely on limited sample numbers, often presenting data from single specimens or representative images, which restricts assessment of inter-individual variability and lacks appropriate controls. Variations in post-mortem interval, sample preparation, and management of autofluorescence are rarely standardised across studies and may influence reported glycan patterns. Donor age is also likely to be a significant factor, as glycosylation is dynamically regulated during development and aging [10]. Despite these limitations, lectin- and antibody-based analyses provide a valuable foundation for defining the retinal glycan nanolandscape and guiding more comprehensive, quantitative studies.

### Endogenous lectins remain poorly understood

There is a growing appreciation of endogenous lectins on various cell types, but highly enriched on immune cells and for their functions in glycoconjugate recognition in development and homeostasis. Lectins such as Siglecs (sialic acid-binding Ig superfamily lectins) are expressed on microglia. These include Siglec-3, -9, and Siglec-11, the latter being uniquely expressed on human microglia [60]. These are currently under investigation as potential therapeutic targets for macrophage inhibition in macular degeneration [61]. Much remains to be discovered with regard to the role of endogenous lectins on various retinal cell types [62], including galectin-1 and -3 in the RPE [63].

## Glycans in retinal disease

### Retinitis pigmentosa

RP is a group of inherited retinal degenerative disorders characterised by progressive photoreceptor loss and consequent vision impairment [64]. Among the molecular mechanisms implicated in RP, defects in protein glycosylation, more specifically N-glycosylation of rhodopsin have been directly linked to disease pathogenesis [29,30]. Human rhodopsin contains two N-glycosylation sites at the N-terminus (N2 and N12), of which glycosylation at N12 is essential for normal protein function, whereas N2 is dispensable for photoreceptor viability [30]. Notably, mutant rhodopsin lacking N-glycosylation is still correctly synthesised and trafficked to the photoreceptor outer segment, indicating that glycosylation is not required for protein localisation [30]. However, retinal degeneration occurs following light exposure in the N12 mutant, suggesting that N-glycosylation at this site is critical for maintaining rhodopsin stability and/or efficient phototransduction [27,29,30]. These studies were conducted on transgenic *X. laevis* expressing human rhodopsin and only investigated occupancy not altered N-glycosylation. However, they are the few mechanistic studies to highlight a specific and functionally important role for glycosylation in photoreceptor biology and underscore its potential contribution to retinal disease.

### Age-related macular degeneration

AMD is a progressive retinal disease that affects the macula, the central region of the retina responsible for sharp vision, leading to gradual loss of central vision through degeneration of the RPE, photoreceptors, and underlying tissues. In AMD, overall sialic acid levels in the neural retina and RPE are largely unchanged; however, a marked, spatially restricted reduction occurs at the Bruch’s membrane/choroid interface [22], where enrichment seen in healthy tissue is lost, resulting in levels comparable to other retinal layers. The mechanisms underlying this reduction remain unclear but may involve altered sialyltransferase or sialidase activity and changes in local glycoconjugates.

Given that terminal sialic acid is associated with the regulation of complement activation and modulation of immune cell responses, its loss at the Bruch’s membrane/choroid interface may contribute to AMD pathology through dysregulated complement signalling and microglial activity [11]. This is supported by findings from sialic acid–deficient mouse models, which exhibit altered immune responses and retinal dysfunction [14]. Although the direct involvement of sialoglycans in complement factor H interactions remains debated, the observed depletion of sialic acid highlights a potential role for the retinal ‘sialome’ in maintaining immune homeostasis. These findings also support the rationale for emerging therapeutic strategies targeting sialylation, including the use of polysialic acid-based nanoparticles [61,65,66].

In parallel, alterations in other glycan classes have been implicated in AMD progression. Individuals with early and intermediate AMD show increased levels of highly sulphated HS, which has been associated with enhanced lipoprotein retention and may contribute to drusen formation [32]. Notably, the increase in negatively charged HS presumably occurs alongside a reduction in sialic acid at the Bruch’s membrane interface, raising the possibility of compensatory remodelling of the extracellular glycan landscape to maintain overall charge balance [22,32]. Whether this reflects an adaptive response or contributes further to disease progression remains to be determined. Together, these findings highlight coordinated but poorly understood changes in glycosylation at the Bruch’s membrane/choroid interface and underscore the importance of considering multiple glycan classes in the context of retinal disease.

### Diabetic retinopathy

Glycosylation changes are implicated in DR, a progressive microvascular complication of diabetes characterised by chronic vascular dysfunction, including increased capillary permeability and blood–retinal barrier breakdown, leading to retinal lesions and vision loss [67]. Tear fluid, a readily accessible and non-invasive sample, has been used to identify glycan biomarkers, revealing approximately 50 N-glycan structures, most of which are fucosylated and contain bisecting N-acetylglucosamine [68]. O-glycan analysis similarly revealed a predominance of core 2 structures [68]. However, only a small subset of low-abundance glycans showed significant changes in individuals with diabetes or DR, and the protein-specific origins of these alterations remain to be established [68]. Complementary studies examining tear GAGs reported increased overall GAG concentrations in patients with both background and proliferative DR, suggesting broader alterations in extracellular glycan composition in disease [69]. Given the ease and non-invasive nature of tear collection, comprehensive glycomic profiling and glycoproteomics of tears could offer significant opportunities for biomarker development and disease stratification [70].

Changes in glycosylation are also evident in the vitreous humour, where an increase in total N-glycans and a shift towards greater sialylation have been observed in DR [71]. *In vitro* models suggest that elevated glucose conditions can drive these changes, with human retinal microvascular endothelial cells (HRMECs) exhibiting increased expression of sialyltransferases, including ST3GAL1 and ST3GAL4, under hyperglycaemic conditions [71]. Suggesting altered sialylation pathways as a potential contributor to disease-associated vascular dysfunction.

Intracellular glycosylation, O-GlcNAc modification, has emerged as a key regulator of retinal vascular pathology [50]. O-GlcNAcylation is markedly elevated in retinal endothelial cells under diabetic conditions, driven by increased glucose availability and hypoxic stress [52]. Functional studies using HRMECs and animal models demonstrate that inhibition of O-GlcNAc transferase can mitigate vascular dysfunction, highlighting its pathological relevance [52]. Mechanistically, O-GlcNAcylation of the transcriptional coactivators YAP and TAZ enhances their stability and activity by suppressing phosphorylation, promoting a pro-angiogenic transcriptional programme. This process is further reinforced by a positive feedback loop with the hexosamine biosynthesis pathway, linking metabolic dysregulation to aberrant vascular signalling. Together, these findings identify O-GlcNAcylation as a critical mediator of blood–retinal barrier breakdown and pathological angiogenesis in DR [52]. In combination with evidence implicating altered sialylation in disease-associated vascular dysfunction, these mechanistic studies suggest that glycosylation pathways may represent promising therapeutic targets. However, further validation in large diverse patient cohorts and a more comprehensive understanding of the underlying mechanisms will be required before these findings can be translated into clinical practice.

## Conclusions

The retina has a remarkable spatially organised landscape of distinct glycan patterns spanning cell surface glycoproteins, glycolipids, and intracellular O-GlcNAcylation. The conservation of these patterns across species suggests fundamental roles for glycans in retinal function and vision. However, the field is lacking clear understanding of the specific role of such glycan patterns during ontogeny and organ homeostasis.

While glycosylation is known to regulate protein stability, turnover, molecular recognition, signalling, extracellular environment, and immune processes, these roles have only been explored in limited contexts in the retina. A major gap is the absence of comprehensive glycoproteomic studies capable of resolving site-specific glycan microheterogeneity. Most current analyses focus on glycosylation site occupancy or broad glycan classes, overlooking the fine structural variation likely to underpin functional specificity, such as linkage of sialic acid, core or terminal fucose, etc. Importantly, our ability to monitor retinal glycans in living patients remains limited, with vitreous sampling providing only a highly invasive window into adjacent retinal glycosylation.

Despite their therapeutic potential, glycans remain challenging targets because their expression is not directly template-driven. Glycan structures are determined by the combined activities of glycosyltransferases and glycosidases, substrate availability, and cellular metabolism, meaning that changes in gene expression do not necessarily predict glycan abundance or composition. Furthermore, many glycan motifs are shared among multiple cell types, presenting an additional challenge for the development of cell-specific glycan-based diagnostics and therapeutics. Nevertheless, advances in other fields, particularly oncology, suggest that combining glycan recognition/blocking strategies with more conventional approaches, utilising dual-targeting systems incorporating both lectins and antibodies, may improve outcomes [72]. Similar approaches could eventually enable the targeting or blocking of disease-associated glycosylation changes in specific retinal cell populations.

The highly organised architecture of the retina further highlights the importance of spatial context in glycosylation. Layer and cell-specific glycan signatures suggest that even subtle compositional changes may have significant functional consequences in development, neural connectivity, and disease. Emerging approaches, particularly multi-omic mass spectrometry imaging, offer significant potential to address this by enabling spatially resolved, integrated analyses of glycans alongside proteins, lipids, and mRNA.

Insights from the broader central nervous system, where glycans regulate synaptic plasticity, neuroinflammation, and neurodegeneration, underscore their likely importance in retinal biology [73,74]. Extending these findings to the retina represents a key opportunity for future research. Although retinal organoids are an immensely useful model system [75], organoids in general can have remarkably different glycosylation and therefore should be tested and taken into consideration.

We acknowledge that glycans are not necessarily the drivers of disease but hypothesise that they are changed during retinal disease and possibly contribute to dysregulated inflammation. Given how little is currently known about the retinal glycome, a stronger descriptive foundation will be crucial for guiding future mechanistic studies and ultimately translating glycobiology into clinical applications for retinal disease.

## Perspectives

- Glycans are essential for a plethora of functions in the human body, yet our current understanding of glycosylation of retinal cells remains very limited.
- Cell types within the retina are differentially glycosylated, resulting in spatial layering of glycan motifs. Dysregulation of glycosylation has been associated with many retinal diseases, including DR, AMD, and RP.
- There are many avenues for future direction, including glycoproteomic and spatial multi-omic analysis. Descriptive studies are still needed to move towards functional studies to clearly articulate the role of glycans in vision and disease.

## Open Access

Open access for this article was enabled by the participation of La Trobe Universiy in an all-inclusive Read & Publish agreement with Portland Press and the Biochemical Society under an agreement with CAUL.

## Abbreviations

AMD: age-related macular degeneration
CS: chondroitin sulphate
DR: diabetic retinopathy
DS: dermatan sulphate
ER: endoplasmic reticulum
EndoN: endoneuraminidase
GAGs: glycosaminoglycans
GSLs: glycosphingolipids
HS: heparan sulphate
HRMECs: human retinal microvascular endothelial cells
HA: hyaluronan
IPM: interphotoreceptor matrix
KS: keratan sulphate
NF-κB: nuclear factor-kappa B
OGA: O-GlcNAcase
OGT: O-GlcNAc transferase
PNA: peanut agglutin
RDS: retinal degeneration slow
RPE: retinal pigment epithelium
RP: Retinitis pigmentosa
